# Special Issue: Damage Analysis for Composite Materials: Methods, Testing and Evaluation

**DOI:** 10.3390/ma17102314

**Published:** 2024-05-14

**Authors:** Luis M. P. Durão, Nuno C. Loureiro

**Affiliations:** 1 INEGI Inst. de Ciência e Inovação em Eng. Mecânica e Eng. Industrial, R. Dr. Roberto Frias, 400, 4200-465 Porto, Portugal; n.loureiro@doc.isvouga.pt; 2 ISEP, Instituto Politécnico do Porto, R. Dr. António Bernardino de Almeida, 4249-015 Porto, Portugal; 3 ISVOUGA, Instituto Superior de Entre Douro e Vouga, R. António de Castro Corte Real, Apart. 132, 4520-181 Santa Maria da Feira, Portugal

After the Stone, the Bronze, and the Iron Ages, material history is now in the Composite Age. Composite materials consist of two or more constituent materials with significantly different physical or chemical properties, which, when combined, produce a material with enhanced properties such as high strength, stiffness, and low weight. Thanks to these unique characteristics, composites are widely used in the production of parts for a large number of final products. In the production process of composite parts, circular hole drilling may be necessary to allow their assembly and incorporation into complex sets, using screws, bolts, pins, rivets, or snap springs. These operations are currently carried out by machining, as drilling is widely used. In the drilling process, delamination is the most frequent damage and can reduce the load-bearing capacity of the parts. The optimization of drilling parameters, the development of specific tool geometries, and the consequences in terms of damage caused by machining have been extensively studied. However, there are still some open issues addressing damage analysis and the assessment of the structural integrity outcomes on load-bearing capacity, fatigue behaviour, or other loading conditions, that deserve research and discussion on parts performance. On the whole, damage analysis for composite materials can be considered a multidisciplinary field that combines materials science, mechanics, numerical analysis, and experimental techniques to ensure the safe and efficient utilisation of composite structures in various applications.

This Special Issue on Damage Analysis for Composite Materials aims to bring to the reader some of the most recent advanced contributions on the study of the outcomes of damage in composite parts, including modelling techniques, failure modes, damage initiation and propagation, durability and fatigue analysis, damage tolerance and repair, non-destructive evaluation, post-failure analysis, and environmental concerns, representing the diverse potential approaches to the study of composite materials. As Guest Editors, it was an honour to be able to bring out all of these contributions in the form of original research articles, showing the ongoing work to broaden the knowledge on this subject.

We cordially invite you to read the papers as they are an interesting set of contributions for the discussion on the subject of damage analysis for composite materials. In [1], the focus was on the interfacial shear strength of carbon nanotubes using finite element methods together with molecular dynamics (MD) and molecular mechanics (MM) to study the interfacial shear strength (ISS) of a structure formed by a pristine single-walled CNT (SWCNT) inserted in a tobermorite crystal. An experimental study on the fracture behaviour of repaired honeycomb/carbon–epoxy sandwich panels under edgewise compression and three-point bending loading was analysed in [2] and complemented by a three-dimensional finite element analysis incorporating a mixed-mode I + II + III cohesive zone model for estimating the fracture behaviour of sandwich panel repairs. In [3], a newly developed failure theory was applied to predict the loads that would result in the fracture of test specimens with holes manufactured from polylactic acid (PLA), subjected to uniaxial loading, and specimens made of polycarbonate (PC), subjected to combined loading. The assessment of the damage caused by drilling holes using enhanced radiography and the consequent outcomes on the fatigue resistance by the cyclic loading of carbon/epoxy plates with the purpose of establishing an association between the damaged region and the material’s fatigue is presented in [4]. The fatigue failure of carbon/epoxy composites is the focus in [5], proposing an entropy-based failure criterion to investigate the fatigue lifetime of unidirectional CFRPs subjected to multiple-amplitude cyclic loadings. Due to the heterogeneity of CFRPs, a micro-finite element model that considers matrix resin and fibres independently is developed. Non-destructive techniques are used in [6] to investigate the damage mechanism and energy absorption characteristics of E-glass laminates and sandwich structures with GFRP face sheets with PVC cores under quasi-static indentation with conical, square, and hemispherical indenters with acoustic emission. Afterwards, a postmortem damage assessment was performed with X-ray micro-computed tomography and scanning electron microscopy. In [7], the focus is on showing that Projection-based Digital Volume Correlation (P-DVC) allows 4D (i.e., space and time) full-field measurements that should be carried out over entire loading histories, enabling the quantification of damage detection and growth over the entire loading history up to failure in a polymer matrix composite. Environmental concerns are presented in [8] on a study of the mechanical outcomes due to machining by flexural testing, showing that the use of alternative formulations with micro-inclusions from recovered waste can contribute both to the reduction in the mechanical degradation of composites and to the environment by avoiding the increase in landfill waste. In [9], the Kolmogorov–Sinai metric entropy is used to determine the transition from the elastic to the viscoelastic state in GFRP composite materials during a static tensile test. Additionally, the acoustic emission method is used, showing that it is possible to determine additional parameters affecting the strength of the structure for any composite materials. Finally, in [10], experimental and finite-element simulation are used to study the stability and failure phenomena of thin-walled constructions subjected to axial compression, featuring a central cut-out, and constructed from composite materials. The comparison underscored a significant concordance between the simulation predictions and the empirical findings.

Since November 2022, this Special Issue has attracted the interest of respected researchers from all over the world. The success of this Issue is evidenced by the publication of 10 papers that underwent a rigorous review process conducted by experts in the field. The Guest Editors would like to congratulate all of the authors of the published works and also thank the reviewers for their time and very valuable comments and suggestions that raised the rank and substantive value of this Special Issue. The success of this Special Issue would not be possible without the constant contact and kind support of the Section Managing Editor, Ms. Serena Shi, who should be gratefully thanked for her dedication and commitment. The Guest Editors also thank the Editors-in-Chief of *Materials* for this opportunity to collaborate on the journal and congratulate them on their stewardship of a globally respected journal. The diligence, creativity, and friendly and dynamic cooperation of all those mentioned above contributed to the success of this Special Issue.
